# [18F]FLT PET for Non-Invasive Assessment of Tumor Sensitivity to Chemotherapy: Studies with Experimental Chemotherapy TP202377 in Human Cancer Xenografts in Mice

**DOI:** 10.1371/journal.pone.0050618

**Published:** 2012-11-30

**Authors:** Mette Munk Jensen, Kamille Dumong Erichsen, Fredrik Björkling, Jacob Madsen, Peter Buhl Jensen, Maxwell Sehested, Liselotte Højgaard, Andreas Kjær

**Affiliations:** 1 Cluster for Molecular Imaging, Faculty of Health and Medical Sciences, University of Copenhagen, Copenhagen, Denmark; 2 Department of Clinical Physiology, Nuclear Medicine & PET, Rigshospitalet, Denmark; 3 TopoTarget A/S, Symbion Science Park, Copenhagen, Denmark; Cincinnati Children’s Hospital Medical Center, United States of America

## Abstract

**Aim:**

3′-deoxy-3′-[^18^F]fluorothymidine ([18F]FLT) is a tracer used to assess cell proliferation *in vivo*. The aim of the study was to use [18F]FLT positron emission tomography (PET) to study non-invasively early anti-proliferative effects of the experimental chemotherapeutic agent TP202377 in both sensitive and resistant tumors.

**Methods:**

Xenografts in mice from 3 human cancer cell lines were used: the TP202377 sensitive A2780 ovary cancer cell line (n = 8–16 tumors/group), the induced resistant A2780/Top216 cell line (n = 8–12 tumors/group) and the natural resistant SW620 colon cancer cell line (n = 10 tumors/group). *In vivo* uptake of [18F]FLT was studied at baseline and repeated 6 hours, Day 1, and Day 6 after TP202377 treatment (40 mg/kg i.v.) was initiated. Tracer uptake was quantified using small animal PET/CT.

**Results:**

TP202377 (40 mg/kg at 0 hours) caused growth inhibition at Day 6 in the sensitive A2780 tumor model compared to the control group (P<0.001). In the A2780 tumor model TP202377 treatment caused significant decrease in uptake of [18F]FLT at 6 hours (-46%; P<0.001) and Day 1 (-44%; P<0.001) after treatment start compared to baseline uptake. At Day 6 uptake was comparable to baseline. Treatment with TP202377 did not influence tumor growth or [18F]FLT uptake in the resistant A2780/Top216 and SW620 tumor models. In all control groups uptake of [18F]FLT did not change. Ki67 gene expression paralleled [18F]FLT uptake.

**Conclusion:**

Treatment of A2780 xenografts in mice with TP202377 (single dose i.v.) caused a significant decrease in cell proliferation assessed by [18F]FLT PET after 6 hours. Inhibition persisted at Day 1; however, cell proliferation had returned to baseline at Day 6. In the resistant A2780/Top216 and SW620 tumor models uptake of [18F]FLT did not change after treatment. With [18F]FLT PET it was possible to distinguish non-invasively between sensitive and resistant tumors already 6 hours after treatment initiation.

## Introduction

A challenge during development of new anti-cancer drugs is to discriminate between responders and non-responders early in the course of treatment. During pre-clinical development of anti-cancer agents testing of in-vivo effect of new drug-candidates with imaging biomarkers can help in guidance of which drug candidates to further develop, improve knowledge of drug candidates and help in selecting which predictive biomarkers could be included in future clinical studies. Many new and already approved chemotherapeutics do only have anti-tumor effect in a subgroup of patients. Identification of these patients early following treatment start could result in a shift toward other treatments in the non-responding patients and consequently reduce unnecessary treatments. The use of the non-invasive positron emission tomography (PET) imaging technique to image cell-proliferation with the tracer 3′-deoxy-3′-[18F]fluorothymidine ([18F]FLT) has been tested in different pre-clinical settings [1]–[10]. [18F]FLT is used to assess cell proliferation *in vivo* by PET, by measuring the activity of thymidine kinase 1 (TK1) which is up-regulated in the S-phase of cell cycle [11]–[16]. TK1 is an enzyme of the DNA salvage pathway. TK1 converts thymidine into thymidine monophosphate (whereby it is further phosphorylated into thymidine triphosphate and incorporated in DNA) and thereby have a key function in DNA syntheses and cell proliferation. TK1 is a central enzyme involved in the uptake of [18F]FLT and therefore measurements of TK1 gene expression was included in the present studies for evaluation of [18F]FLT uptake. The standard method for assessment of cell proliferation in tumors is by Ki67 immunohistochemistry. Measurement of the amount of Ki67 positive cells in a solid tumor requires a biopsy and therefore sequential measurements of cell proliferation in tumors during treatment is often limited because of the challenges with removal of serial biopsies. Ki67 antigen is a protein expressed in proliferating cells where it is located in the nucleolus [17]. Ki67 is expressed in G1, S, G2 and M phase of cell cycle but not during the resting G0 phase, the expression being highest in the mitotic phase [18]. Cell proliferation is often either the primary or secondary target of many anti-cancer drugs, and therefore investigation of changes in cell proliferation by use of [18F]FLT PET can be used following treatment with various anti-cancer drugs. However, [18F]FLT changes following treatment are very variable and dependent on the tumors and treatments [19].

Even though many pre-clinical studies have investigated changes in [18F]FLT uptake following treatment with different chemotherapeutics few studies have analyzed differences in uptake of [18F]FLT between responding and non-responding tumors [20], [21].

**Figure 1:**
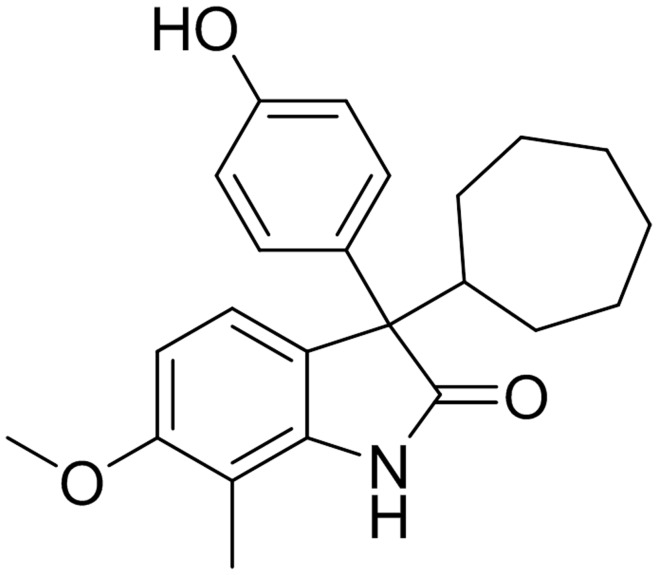
Chemical structure of TP202377.

Previously, we found that [18F]FLT decreased 2, 6 and 24 hours following treatment with Top216 in a sensitive A2780 tumor model [22]. TP202377 is an analogue of the previously described Top216 with the same potent anti-tumor activity but lower toxicity [23]. Both Top216 and TP202377 belong to a compound group build on a 1,3-dihydroindole-2-one scaffold. These compounds inhibit protein and DNA synthesis and induce apoptosis and show potent anti-cancer activity in several cell lines and mouse models of human cancer. TP202377 induces complete tumor regression in a rat PC3 (human prostate cancer cell line) xenograft model [23]. The exact mechanism of action of these compounds is still unknown; however, the anti-cancer effect is somehow linked to the mTOR pathway and it might be due to depletion of intracellular amino acids [24].

In order to investigate if the decrease in [18F]FLT uptake following treatment is predictive for a later regression in tumor size, there is a need for knowledge about if the early changes in [18F]FLT uptake are specific for the sensitive tumors. In this study we investigated [18F]FLT uptake in three tumor models of which two are resistant to TP202377 (A2780/Top216 and SW620) [23], [24] following treatment in order to investigate if the changes in [18F]FLT uptake reveals whether tumors are drug sensitive. We used the sensitive A2780 ovary tumor model because TP202377 has anti-tumor effect in this tumor model and development of new chemotherapeutics for treatment of ovarian cancer is highly needed. Many ovarian cancer patients show an initial response to chemotherapy; however, numerous patients relapse with drug-resistant tumors and therefore development of new chemotherapeutics for treatment of ovarian cancer is of great interests.

The aim of the study was therefore to analyze if [18F]FLT PET could be used to separate responding from non-responding tumors within 24 hours following treatment start with the new anti-cancer compound TP202377. To do so, we imaged cell proliferation *in vivo* with [18F]FLT PET before and during treatment in both a TP202377 sensitive and two resistant human cancer mouse tumor models. The imaging data were compared with Ki67 and TK1 gene expression and tumor growth measured with computed tomography (CT).

**Table 1:** Primer sequences for Ki67, TK1 and reference genes.

Gene	Forward primer (5′-3′)	Reverse primer (5′-3′)	Amplicon length (bp)
Ki67	tcccgcctgttttctttctgac	ctctccaaggatgatgatgctttac	121
TK1	gccgatgttctcaggaaaaagc	gcgagtgtctttggcatacttg	103
PPIA	cggatttgatcatttggtg	cagggaatacgtaaccag	123
RPLP0	ctgtggagacggattac	ggcttcaaccttagctg	146
TBP	ggaagacgacgtaatgg	ctgcaactcaacatcca	130

## Materials and Methods

### Tumor Model

Animal care and all experimental procedures were performed under the approval of the Danish Animal Welfare Council (2006/561-1124). Female NMRI (Naval Medical Research Institute) nude mice (8 weeks old) were acquired from Taconic Europe (Lille Skensved, Denmark) and allowed to acclimatize for one week in the animal facility before any intervention was initiated. Tree different cell lines were used, the TP202377-sensitive human ovarian carcinoma cell line A2780 [25] (a gift from R. Ozols, Fox Chase Cancer Center Philadelphia, PA, January 2004), a TP202377-resistant form of the A2780 cell line A2780/Top216 and the human colon cancer cell line SW620. The SW620 cell line was acquired from ATCC (LGC Standards AB, Borås, Sweden). The Top216 resistant A2780 clone (A2870/Top216) was made after serial *in vitro* passages and clone selection of A2780 in the presence of increasing concentrations of Top216. The A2780/Top216 clone is cross-resistant to TP202377 [23].

**Figure 2:**
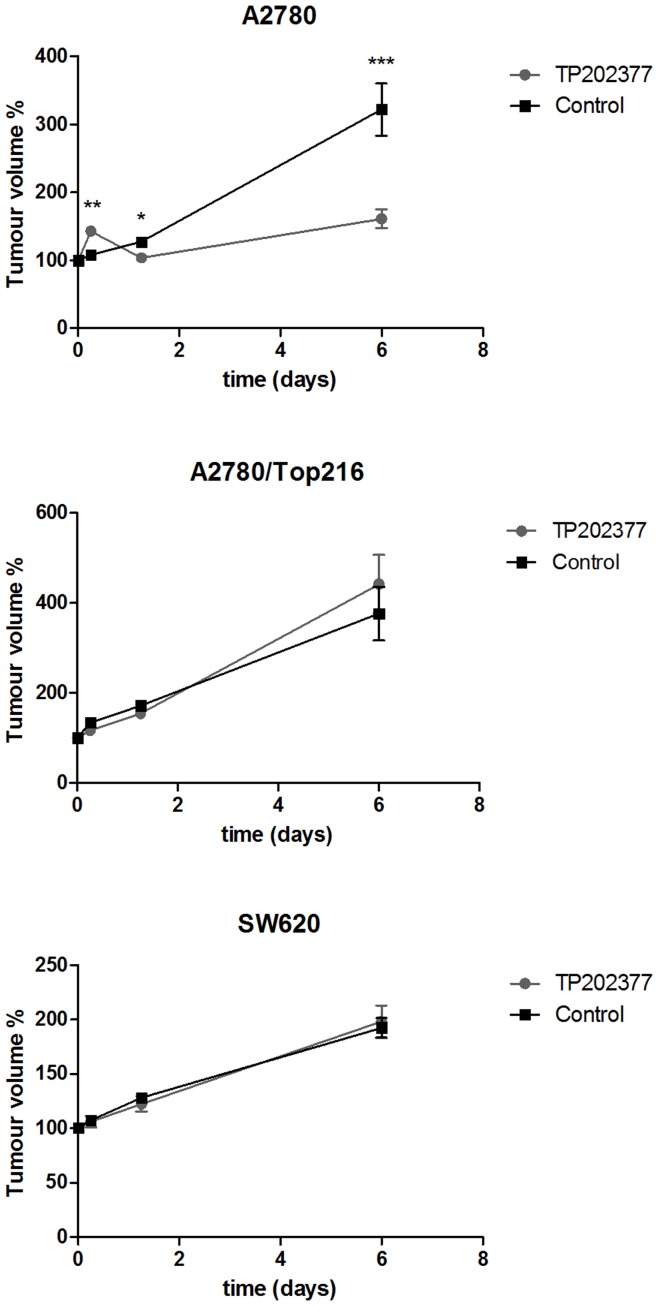
Tumor volume. Growth of tumors following TP202377-treatment of A2780 (top panel), A2780/Top216 (mid panel) and SW620 (lower panel). Tumor volume was determined by microCT. The mice were treated with TP202377 (40 mg/kg i.v) or vehicle at Day 0. N = 8–16 tumors/group. *) p<0.05, **) p<0.01, ***) p<0.001 treatment versus control group at same time point.

**Figure 3:**
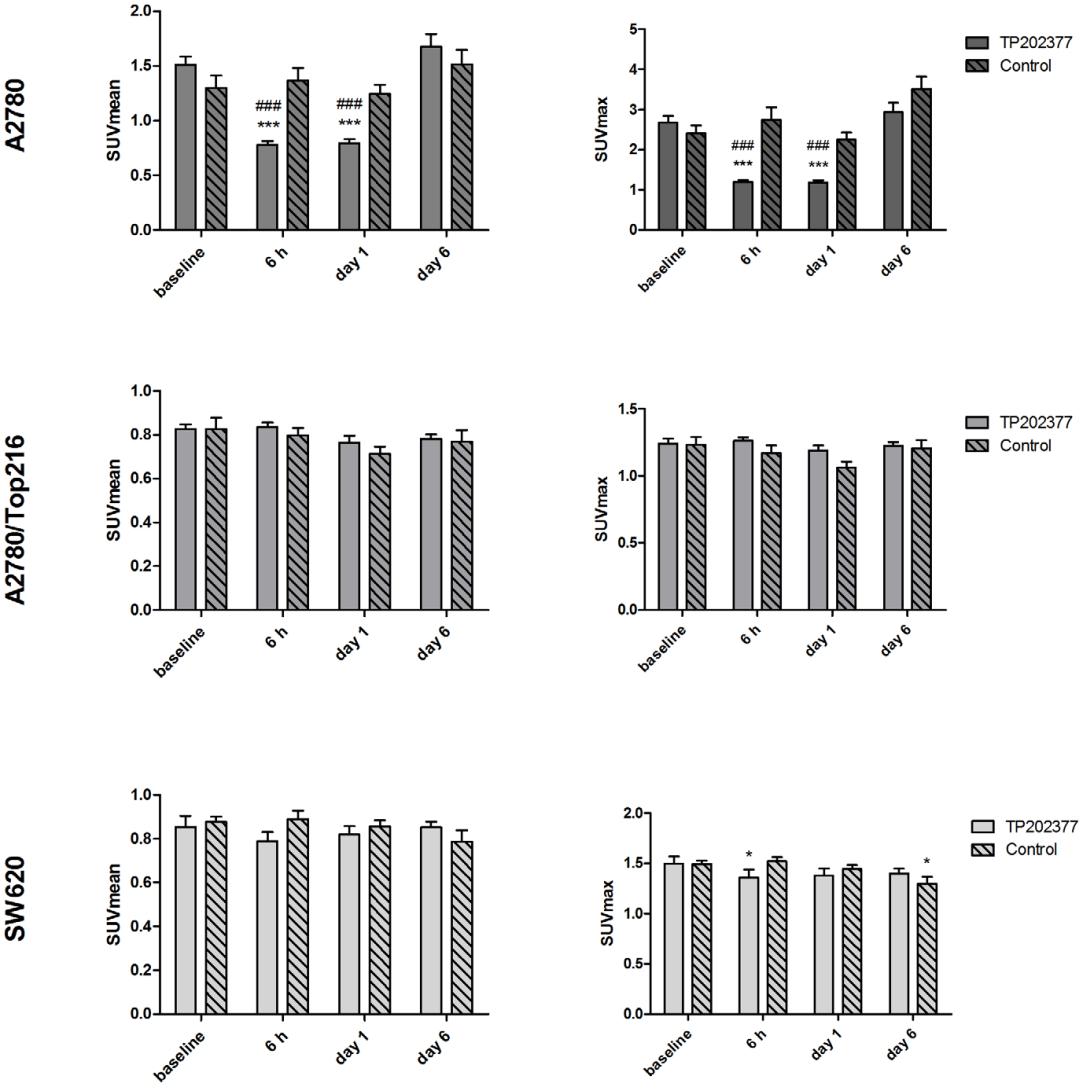
[18F]FLT uptake following treatment. [18F]FLT uptake measured as SUVmean (left panels) and SUVmax (right panels) in A2780, A2780/Top216 and SW620 following treatment with TP202377 or vehicle. N = 8–16 tumors/group. *) p<0.05, **) p<0.01, ***) p<0.001 versus baseline in same treatment group. #) p<0.05, ##) p<0.01, ###) p<0.001 treatment versus control group at same time point.

**Figure 4:**
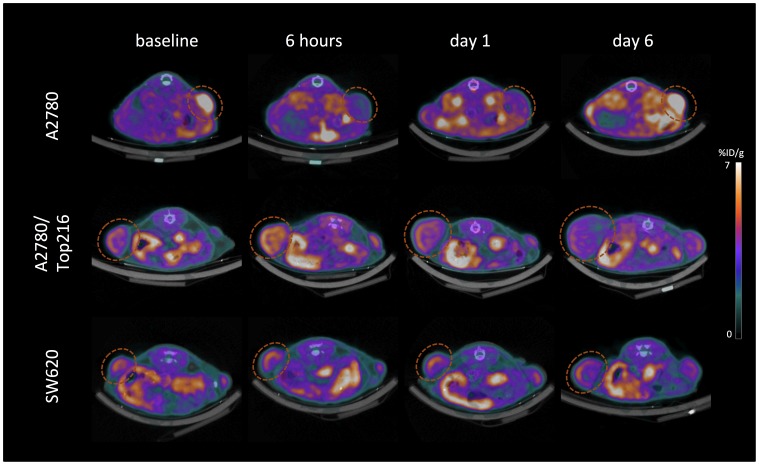
PET/CT images. Representative [18F]FLT PET/CT images of A2780 (upper panel), A2780/Top216 (mid panel) and SW620 (lower panel) xenografts (dotted circles). [18F]FLT uptake is measured in the same animals at baseline and 6 hours, Day 1 and Day 6 follow treatment initiation with TP202377.

**Figure 5:**
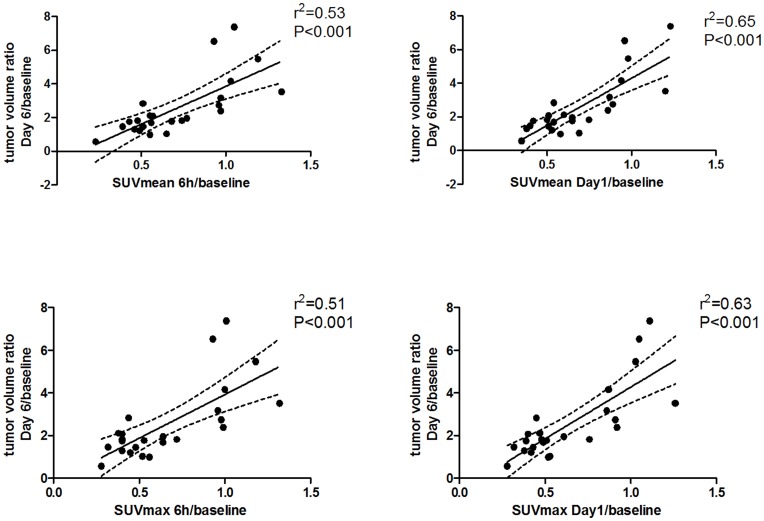
Correlations between changes in [18F]FLT uptake and tumor growth. Changes in tumor volume measured as ratio Day 6/baseline compared with uptake of [18F]FLT measured as ratio of 6 hours/baseline (right panel) and Day 1/baseline (left panel).

**Figure 6:**
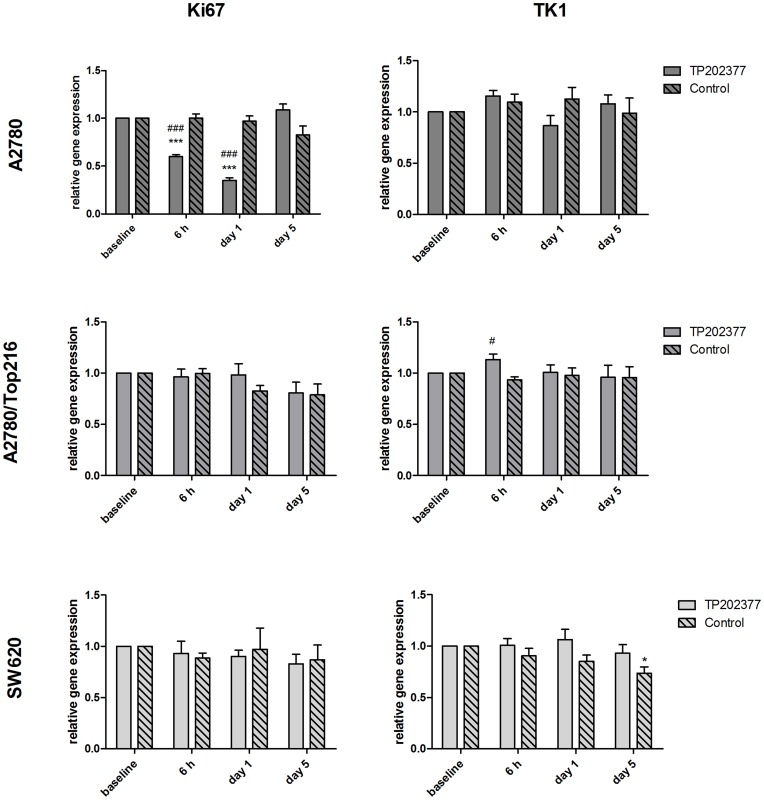
Ki67 and TK1 gene expression. Expression of Ki67 and TK1 normalized to the geometric mean of three reference genes. Data are presented as fold changes following treatment relative to baseline levels. N = 6–8 tumors/group. *) p<0.05, **) p<0.01, ***) p<0.001 versus baseline in same treatment group. #) p<0.05, ##) p<0.01, ###) p<0.001 treatment versus control group at same time point.

For establishment of xenografts, 10^7^ cells in 100 µL medium mixed with 100 µL Matrixgel™ Basement Membrane Matrix (BD Biosciences, San Jose, CA, USA) were injected subcutaneously into the left and right flank respectively during anesthesia with 1∶1 v/v mixture of Hypnorm® (Janssen Pharmaceutica, Beerse, Belgium) and Dormicum® (Roche, Basel, Switzerland). The cell lines has been tested free of mycoplasma. All cell lines were cultured in RPMI (Roswell Park Memorial Institute) medium 1640+ GlutaMAX (Invitrogen, Carlsbad, CA, USA) supplemented with 10% fetal calf serum (Biological Industries, Israel) and 1% penicillin-streptomycin (Invitrogen) in 5% CO_2_ at 37°C.

### Experimental Design

Xenografts in mice from 3 human cancer cell lines were used: the TP202377-sensitive A2780 ovary cancer cell line (n = 4–8 mice/group = 8–16 tumors/group), the induced TP202377-resistant A2780/Top216 cell line (n = 4–6 mice/group = 8–12 tumors/group) and the natural TP202377-resistant SW620 colon cancer cell line (n = 5 mice/group = 10 tumors/group). *In vivo* uptake of [18F]FLT was studied at various time points after treatment with TP202377 (40 mg/kg i.v. at 0 hours) or vehicle (2% DMSO, 20% HP-b-CD in saline). The chemical structure of TP202377 is shown in figure 1. TP202377 was in preceding analyses shown to inhibit growth of the A2780 xenograft tumors but not of the A2780/Top216 and SW620. [18F]FLT scans were performed before either TP202377 or vehicle was injected to determine the baseline level of tracer uptake. The design of the study was longitudinal and the [18F]FLT scans were repeated in the same animals 6 hours, Day 1, and Day 6 (5 to 7) post injection.

Tumor volume was followed by CT during the experiments [26]. Tumor volumes were calculated relative to volume at baseline.

Expression of Ki67 and TK1 were analyzed *in vitro* in parallel groups of mice with A2780, A2780/Top216 and SW620 tumors respectively, which were treated with either TP202377 or vehicle and biopsied before and at 6 hours, Day 1 and Day 5 after initiation of treatment. Biopsies were taken using an 18G needle and placed immediately in RNA later® (Ambion (Europe) Limited, Cambridgeshire, UK). All samples were stored at 4°C and the following day RNAlater® was removed and samples transferred to −80°C until further qPCR processing.

### Synthesis of [18F]FLT

[18F]FLT was synthesized using 3-N-Boc-1-[5-O-(4,4′-dimethoxytrityl)-3-O-nosyl-2-deoxy-β-D-lyxofuranosyl]thymine as precursor and synthesized on a GE TracerLab MX Synthesizer as previously described [22]. All reagents and [18F]FLT cassettes were purchased from ABX (Radeberg, Germany).

### microPET and microCT Imaging

The mice were injected i.v. with 9.6±1.7 (mean±SD) MBq [18F]FLT. One hour after tracer injection mice were anaesthetized with 3% sevofluran (Abbott Scandinavia AB, Solna, Sweden) mixed with 35% O_2_ in N_2_ and fixed on a bed in presence of three fiducial markers allowing fusion of PET and CT pictures. A PET scan was acquired using a MicroPET Focus 120 (Siemens Medical Solutions, Malvern, PA, USA) followed by a microCT scan acquired with a MicroCAT® II system (Siemens Medical Solutions) as previously described [22]. After data acquisition, PET data were arranged into sinograms and subsequently reconstructed with the maximum a posteriori (MAP) reconstruction algorithm. The pixel size was 0.866×0.866×0.796 mm and in the center field of view the resolution was 1.4 mm full-width-at-half-maximum.

PET and microCT images were fused in the Inveon software (Siemens Medical Solutions). Before fusion region of interests (ROIs) were drawn on the CT pictures manually by qualitative assessment covering the whole tumors and subsequently tumor volume and tracer uptake, assessed by standard uptake values (SUV) mean and maximum, were generated by summation of voxels within the tomographic planes.

### Quantitative Real-time Polymerase Chain Reaction (qPCR)

Total RNA was isolated from the biopsies with TRI reagent® following the manufacturer’s instructions (Molecular Research Center Inc., OH, USA) and subsequently RNA integrity was measured on a 2100 Bioanalyzer (Agilent Technologies, Santa Clara, CA, USA). RNA quality is stated as RNA integrity number (RIN). The concentration of the RNA was determined by NanoDrop 1000 (Thermo Fisher Scientific, Wilmington, DE, USA). Total RNA (0.3 µg) was reversed transcribed using the Affinityscript™ QPCR cDNA Synthesis kit (Stratagene, La Jolla, CA, USA) according to the manufacturer’s instructions. Samples were cooled down and stored at -20°C until further use.

Gene expression was quantified on a Mx3000P® real**-**time PCR system from Stratagene. All gene of interests (GOIs) and reference genes were quantified with Brilliant® SYBR® Green QPCR Master Mix (Stratagene). The following thermal profile was used in all experiments: 10 minutes of denaturation at 95°C followed by 45 cycles of 30 seconds denaturation at 95°C, 1 minute of annealing at 60°C and 1 minute extension at 72°C. A dissociation curve was afterward acquired by denaturation of the products for 1 minute at 95°C followed by a stepwise increase in temperature from 55°C to 95°C with steps of 0.5°C/cycle where the duration of each cycle was 18 seconds.

QPCR data were analyzed in the qBase program. The relative quantification of the GOIs was calculated using multiple reference genes [27]. Tissue from baseline samples served as calibrator. The level of the GOIs was normalized to the geometric mean of three reference genes. The three most stable reference genes were found from a panel of 12 candidates, the human reference gene panel (TATAA Biocenter AB, Göteborg, Sweden) by use of the geNorm algorithm.

Primers were designed using Beacon Designer (PREMIER Biosoft, Palo Alto, CA, USA) and the sequences are shown in table 1. For each gene the optimal primer concentration was found. All assays were optimized to have efficiencies between 95% and 105%. All samples were run in triplicate using one µl of cDNA. To each sample a no-reverse transcription control (NoRT) was included, and on each plate a no-template control (NTC) was included.

### Statistical Analysis

Comparisons of tumor volume between treatment and control groups were calculated using unpaired student’s t-test. Paired t-test was used for intra-group comparisons. Bonferroni correction of P-values for multiple comparisons was applied. Correlations between SUVmean and SUVmax ratios and tumor growth were calculated using linear regression. Calculations were made in PASW 18.0 (IBM Corporation, Armonk, New York, USA). Data are reported as mean±SEM and P<0.05 was considered statistically significant.

## Results

### Tumor Volume

A2780 tumors that were treated with TP202377 had volumes at Day 6 that were 161±13% relative to baseline. In the A2780 control group volumes on Day 6 were 322±38% relative to baseline which were significantly higher compared to the treatment group (P<0.001). A2780/Top216 tumors that were treated with TP202377 had volumes at Day 6 that were 442±65% relative to baseline. In the A2780/Top216 control group volumes at Day 6 were 376±59% relative to baseline which was not different from the treatment group. SW620 tumors that were treated with TP202377 had volumes at Day 6 that were 198±15% relative to baseline. In the SW620 control group volumes at Day 6 were 193±9% which was not different from the treatment group (figure 2).

### [18F]FLT Uptake

Baseline uptake of [18F]FLT measured as SUVmean was 1.44±0.06 in the A2780 tumors, 0.83±0.02 in the A2780/Top216 tumors and 0.86±0.03 in the SW620 tumors. In the A2780 tumor model TP202377 treatment caused significant decrease in uptake of [18F]FLT from 1.51±0.07 at baseline to 0.78±0.03 at 6 hours (-46±3%; P<0.001) and 0.79±0.04 at Day 1 (-46±3%; P<0.001) (figure 3). At Day 6 uptake was 1.67±0.12 which was comparable to baseline. Between the treatment and control group the uptake was different at 6 hours (P<0.001) and Day 1 (P<0.001) (figure 4). Treatment with TP202377 did not influence [18F]FLT SUVmean uptake in the resistant A2780/Top216 or SW620 tumor models and no difference between treatment and control groups were observed. In all the control groups [18F]FLT SUVmean did not change during the experiment.

Baseline uptake of [18F]FLT measured as SUVmax was 2.58±0.13 in the A2780 tumors, 1.24±0.03 in the A2780/Top216 tumors and 1.50±0.04 in the SW620 tumors. TP202377 treatment caused significant decrease in uptake of [18F]FLT in the A2780 tumor model as measured by SUVmax. In the treatment group uptake decreased from 2.67±0.17 at baseline to 1.20±0.05 (-53±3%; P<0.001) at 6 hours and 1.19±0.05 (-54±3%; P<0.001) at Day 1. At Day 6 uptake had returned to a baseline level 2.94±0.23. Between the treatment and control group the uptake was different at 6 hours (P<0.001) and Day 1 (P<0.001) (figure 3).

Treatment with TP202377 did not influence [18F]FLT SUVmax uptake in the resistant A2780/Top216 tumor model, however uptake of SUVmax decreased from 1.50±0.07 at baseline to 1.36±0.08 at 6 hours (-10±3%; P = 0.02) in the SW620 tumor model. In the SW620 control group uptake at Day 6 1.49±0.04 was lower compared to baseline uptake 1.29±0.07 (-14±4%; P = 0.04). No difference between the treatment and control groups were observed for either the A2780/Top216 or SW620 tumors (figure 3).

Correlations between SUVmean or SUVmax ratios from baseline to 6 hours and Day 1, respectively, and tumor volume changes from baseline to Day 6 were calculated. For the TP202377 treated A2780 and A2780/Top216 tumors together, we found a significant positive correlation between tumor growth and SUVmean 6 hours/baseline (r^2^ = 0.53; P<0.001), SUVmean Day1/baseline (r^2^ = 0.65; P<0.001), SUVmax 6 hours/baseline (r^2^ = 0.51; P<0.001) and SUVmax Day1/baseline (r^2^ = 0.63; P<0.001) (figure 5).

### Ki67 and TK1 Gene Expression

The tree most stable reference genes were found to be peptidylprolyl isomerase A (PPIA), ribosomal protein P0 (RPLP0) and TATA box binding protein (TBP). The level of the GOIs was normalized to the geometric mean of these three genes. The gene expression levels are stated relative to baseline. RNA integrity numbers (RIN-values) were 8.8±0.9 (mean±SD) for all samples.

Ki67 gene expression was lower in the treatment group compared to the control group at 6 hours (0.60±0.02 vs. 1.00±0.04; P<0.001) and Day 1 (0.35±0.03 vs. 0.97±0.05; P<0.001) after treatment initiation in the A2780 tumor group. At Day 5, expression of Ki67 in the treatment group was comparable to the control group. Compared to baseline expression, Ki67 was decreased at 6 hours (-40±2%; P<0.001) and Day 1 (-65±3%; P<0.001) in the A2780 treatment group. Expression of Ki67 did not change in either of the A2780/Top216 and SW620 treatment groups or any of the control groups.

Expression of TK1 was unchanged in the A2780 tumors in both the treatment and the control group. However, in the A2780/Top216 tumors we observed a small increase in TK1 expression in the treatment compared to the control group at 6 hours (1.13±0.06 vs. 0.93±0.03; P = 0.04) following treatment initiation (figure 6). At Day 5 expression of TK1 was decreased in the SW620 tumor control group compared to baseline (-27±6%; P = 0.02).

## Discussion

The present study demonstrated non-invasive differentiation between responding and non-responding tumors 6 hours after initiation of treatment with the anticancer drug TP202377 by use of [18F]FLT PET. Six hours after injection of TP202377 we observed a -46% decrease in SUVmean values and -53% decrease in SUVmax values in the TP202377-sensitive A2780 tumor type. The decrease in tracer uptake preceded regressions in tumor volume on Day 6. We did not observe changes in SUVmean after treatment of the two TP202377-resistant tumor models. However, in the TP202377-resistant SW620 tumor model we observed a small decrease at -10% 6 hours after injection of TP202377 when measured as SUVmax. At Day 1 SUVmax was comparable to baseline in the SW620 tumors. The reason that we observed this small change in SUVmax uptake at 6 hours could possibly be that this tumor model is not absolute resistant to TP202377 but just has very low drug sensitivity compared to A2780. However, no changes in tumor volume at the end of the experiment were observed despite the small decrease in SUVmax at 6 hours. There was no significant difference in either SUVmean or SUVmax between the treatment and control group 6 hours after treatment start, so we observed still a difference between the responding and non-responding tumors at this time point.

At Day 6 [18F]FLT uptake in the treatment group was comparable to the control group in the sensitive A2780 tumors which indicate that the tumors had started to re-proliferate. We evaluated the [18F]FLT response after a single dose of TP202377 and in order to acquire long term treatment effect the drug needs to be injected several times. Potentially, assessment of a tumors proliferative status with [18F]FLT could be used for determination of the optimal time window between two drug injections.

The changes in [18F]FLT at 6 hours and Day 1 were correlated with tumor volume ratio baseline/Day 6 in order to examine if it, on the individual tumor level, was possible to predict tumor response. There was a good correlation between changes after 6 hours and Day 1 in [18F]FLT tracer uptake and the changes in tumor volume on Day 6. The tumors that decreased most in tracer uptake immediately (6 hours and Day 1) following treatment start were the tumors that later had the lowest increase in tumor volume. Accordingly, 6 hours after treatment start we were able to point out the tumors which responded best to the treatment. During development of new chemotherapeutic drugs, especially in the early clinical phases, identification of responding tumors is of great value in order to predict treatment outcome. Most new anti-cancer treatments have an effect on specific targets in tumors and do in most cases have effect in only subgroups of patients. An identification of the responding patients with [18F]FLT PET early following treatment start might reduce the cost of drug development and avoid unnecessary treatment in patients in whom the therapy does not work as the treatment could be stopped in the non-responding patients. Also, [18F]FLT PET would allow for use of potent drugs that are only effective in few patients if combined with imaging response monitoring.

The level of Ki67 and TK1 gene expression was measured in a parallel study. Expression of Ki67 paralleled the uptake of [18F]FLT, which confirmed the PET results by indicating that the decrease in tracer uptake was due to a real physiologic change and not a consequence of e.g. decreased delivery of [18F]FLT. No changes in TK1 gene expression were observed. In a previous study, where [18F]FLT uptake following treatment with the TP202377 analogue Top216 was analyzed, deviation between [18F]FLT uptake and TK1 expression was observed as well [22]. It seems like treatment with these drugs, despite inducing a significant decrease in [18F]FLT uptake, does not regulate TK1 gene expression and more studies are needed in order to know more about the influence of TK1 on the [18F]FLT uptake following treatment with TP202377. Other studies have similarly not found any changes in mRNA levels of TK1 despite a change in [18]FLT tracer uptake [2] or only observed a week correlation between [18F]FLT and TK1 mRNA [28]. Despite TK1 is being central factor in the uptake of [18F]FLT these two are not always tightly linked [29], and changes in [18F]FLT uptake could be due to changes in protein levels and activity or changes in ATP levels [5], [12], [30], [31].

One of the TP202377-resistant cell lines used in this study was derived from the TP202377-sensitive cell line A2780. The SW620 cell line was previously found to be naturally resistant to TP202377. We used both a induced resistant and a naturally resistant cell line in order to analyze if there would be any difference in the [18F]FLT response to the treatment between the two cell lines. Interestingly, the [18F]FLT uptake in each of the TP202377-resistant cell lines was lower compared to the TP202377-sensitive cell line A2780. However, the uptake of [18F]FLT was in both cell lines above background levels. Low uptake of [18F]FLT in SW620 has also been reported by others [32].

In many studies, the effect of many different kinds of chemotherapy on uptake of [18F]FLT has been studied [1]–[10]. However, few studies have tested both sensitive and resistant tumors. One study analyzed [18F]FLT uptake in both a cetuximab-sensitive and a cetuximab-resistant cell line following treatment with cetuximab; however, no changes in FLT uptake were seen in either of the cell lines [20]. Another study found no discernible effect of trastuzumab treatment on tumor uptake of [18F]FLT in a cohort of both responding and non-responding tumors [21].

In conclusion, we found a -53% decrease in [18F]FLT SUVmax uptake 6 hours after treatment start in the TP202377-sensitive A2780 tumor model. In the TP202377-resistant tumor models we did not see any clinical relevant changes in [18F]FLT uptake following treatment. Tumor growth inhibition after TP202377 treatment was observed in the responding A2780 tumor model but not the two non-responding tumor models. This study demonstrated that differences in [18F]FLT uptake 6 hours after treatment start with a new anti-tumor compound could separate responding from non-responding tumors before any regression in tumor size was observed. We believe this concept hold great promises both for drug development and tailoring cancer therapy.
